# Implementing Remote Radiotherapy Planning to Increase Patient Flow at a Johannesburg Academic Hospital, South Africa: Protocol for a Prospective Feasibility Study

**DOI:** 10.2196/60131

**Published:** 2025-07-28

**Authors:** Duvern Ramiah, Sonwabile Ngcezu, Oluwatosin Ayeni, Okechinyere Achilonu, Mariam Adeleke, Theo Nair, Joseph Otten, Daniel Mmereki

**Affiliations:** 1 Division of Radiation Oncology, School of Clinical Medicine Faculty of Health Sciences University of the Witwatersrand Johannesburg South Africa; 2 School of Clinical Medicine, Medical Physics Faculty of Health Sciences University of the Witwatersrand Johannesburg South Africa; 3 Medical Physics Sefako Makgatho Health Sciences University Pretoria South Africa; 4 Epidemiology and Biostatistics Division, School of Public Health Faculty of Health Sciences University of the Witwatersrand Johannesburg South Africa; 5 Department of Statistical Science University College London London United Kingdom; 6 Varian Siemens Healthiers UK London United Kingdom

**Keywords:** remote radiotherapy planning services, radiotherapy, remote radiotherapy, feasibility, treatment planning, quality assurance, remote treatment, cancer care

## Abstract

**Background:**

Access to timely radiotherapy in resource-constrained environments, particularly low- and middle-income countries (LMIC), is hampered by infrastructure constraints, workforce shortages, and a rising cancer burden. Remote radiotherapy planning (treatment planning as a service [TPaaS]) has the potential to enhance workflow efficiency, reduce wait times, and expand access to treatment. However, its integration and feasibility in LMIC public health systems remain underexplored.

**Objective:**

This study evaluates the feasibility and initial effectiveness of remote radiotherapy planning using the Varian Eclipse system integrated with Elekta Versa HD linear accelerators (LINACs) at the busiest public hospital in South Africa. The primary goal is to determine whether remote planning can maintain plan quality while enhancing efficiency and minimizing treatment delays.

**Methods:**

A prospective, single-site, pilot study is being conducted at Charlotte Maxeke Johannesburg Academic Hospital (CMJAH) in 2 phases. Phase 1 (feasibility) encompasses system commissioning, including beam modeling, computed tomography (CT)-to-electron density calibration, multileaf collimator (MLC) optimization, and dose calculations using the anisotropic analytical algorithm. System performance is validated through gamma index analysis (≥95% pass at 3%/3 mm). Interoperability and workflow readiness are assessed using simulated clinical scenarios and time integration steps. Phase 2 (effectiveness/impact) evaluates operational outcomes in 100 screened adult patients (≥18 years) with cervical, breast, prostate, head and neck, or rectal cancers requiring curative radiotherapy. Patients are grouped by cancer type (25 per group). Time to treatment, plan quality, and system efficiency will be compared with historical in-person planning data. Key workflow metrics include dates of first consultation, CT simulation, planning initiation, plan approval, quality assurance, and treatment start and completion.

**Results:**

The study commenced enrollment in November 2023, with completion anticipated by mid-2025. As of July 2024, approximately 44 patients were screened and are anticipated to complete the remote planning. Initial findings show successful MLC transmission and dosimetric leaf gap optimization through iterative testing. Gamma pass rates exceeded 90% on both clinical and test servers, demonstrating initial accuracy. Results, including planning timelines, quality assurance outcomes, and system performance, will be available following comprehensive analysis in the third quarter of 2025. Preliminary findings indicate effective integration of remote planning in a resource-constrained public health sector setting.

**Conclusions:**

This study shows that remote radiotherapy planning is feasible and might improve cancer treatment in LMIC. The integration of commercially available systems, such as TPaaS, was successfully achieved without compromising dosimetric quality and ensured workflow continuity. Remote planning could serve as an effective tool to reduce treatment delays and enhance resource utilization in oncology units facing high demand. These findings offer valuable insights into technical integration, quality planning, and workflow outcomes, which can inform the future implementation of effective strategies in resource-constrained settings.

**International Registered Report Identifier (IRRID):**

DERR1-10.2196/60131

## Introduction

Research indicates that there is a growing global demand for cost-effective, high-quality [1-4], and sustainable radiotherapy solutions in low- and middle-income countries (LMIC) to address rising cancer cases [5]. However, existing strategies often lack affordability and adequate support, particularly in LMIC, where cancer incidence is high, staff shortages are severe, and health care resources are constrained [6]. Consequently, 55 countries lack radiotherapy, and 80 countries face significant shortages [5]. Even in high-income countries (HIC), centralized radiotherapy networks limit access, particularly in geographically dispersed populations (for example, in Canada, Australia, and the United Kingdom) [7-10].

The quality of radiotherapy relies on accurate treatment planning that optimally balances effective tumor control with the preservation of surrounding healthy tissues [11]. Technological advances over the past two decades have enhanced the precision the delivery and planning of radiotherapy, yet significant challenges remain [12]. In South Africa, a rising cancer incidence, limited radiotherapy equipment, and critical staff shortages contribute to delays ranging from several months to years (ranging from 3 months to 5 years) [13]. Public sector facilities experience prolonged waiting times, underscoring the need for solutions to optimize radiotherapy workflows [14-16], particularly in LMIC [17].

Access to cancer treatment in LMIC is hindered by limited infrastructure, treatment facilities, planning systems [18], and human resources [19,20]. Although advanced modalities, such as intensity-modulated radiotherapy (IMRT), volumetric-modulated arc therapy (VMAT), and emerging therapies [21], offer significant potential, their implementation is often resource-intensive and time-consuming. In Africa, the cancer burden is expected to rise sharply, with new cases projected to increase from 844,000 in 2012 to over 1.5 million in 2030 [19,22], driving a parallel rise in the demand for radiotherapy services. The World Health Organization (WHO) and International Atomic Energy Agency (IAEA) have provided radiotherapy guidelines to support the development and implementation of radiotherapy services globally [23]. However, many African countries lack comprehensive cancer care infrastructure. As of 2020, the continent had only 430 external beam radiotherapy megavoltage units, with over one-half concentrated in Egypt (119 units) and South Africa (97 units) [19]. Addressing radiotherapy needs in such settings necessitates not only physical infrastructure but also trained personnel, robust quality assurance (QA) protocols, and integration of clinical workflows.

Remote treatment planning, including treatment planning as a service (TPaaS), offers a promising strategy to enhance access and reduce delays in radiotherapy delivery in resource-limited environments. Off-site planning enables the redistribution of specialized expertise without requiring patient relocation, thus optimizing service delivery in under-resourced settings. Although remote radiotherapy planning and artificial intelligence (AI)–based systems are increasingly studied in HIC [24-26], limited research has explored their applicability and impact in LIMC, particularly in the South African public sector. A notable exception is the study by McGinnis et al [27], who surveyed radiation oncology providers across sub-Saharan Africa (SSA; including Botswana, South Africa, and Tanzania) and Central America (Guatemala) regarding their experience with the Radiation Planning Assistant, web-based software aimed at improving radiotherapy planning accessibility and quality in LMIC. Despite its promise, the feasibility of remote planning for reducing overall treatment timelines requires further investigation. Therefore, a comprehensive understanding of remote radiotherapy planning’s potential to improve patient flow in LMIC, particularly South Africa, is essential. To evaluate the feasibility of remote radiotherapy planning in resource-constrained settings and commissioning of remote radiotherapy planning, measurement of beams is required.

Given the substantial delays in radiotherapy initiation, ranging from 3 months to 5 years, at Charlotte Maxeke Johannesburg Academic Hospital (CMJAH), the region’s largest public sector radiotherapy center, there is a pressing need for workflow innovations. CMJAH primarily manages cervical, prostate, breast, head and neck, and rectal cancers. Delays are exacerbated by a shortage of radiotherapy machines and a critical shortage of radiotherapists and medical physicists. To date, no studies have evaluated the feasibility of remote radiotherapy planning in this context. Therefore, this study presents the implementation of remote radiotherapy in the Radiation Oncology Division at CMJAH. This study has two primary aims: The first is to (1) evaluate the feasibility of implementing remote radiotherapy planning to improve workflows, with a focus on integrating the Varian Eclipse treatment planning system (TPS) with Elekta Versa HD linear accelerators (LINACs). This includes assessing workflow integration, QA processes, planning system functionality, and analysis of dose calculation data through comprehensive commissioning in a resource-constrained setting. The second is to (2) explore the impact of remote workflows on key operational metrics, such as time-to-treatment, resource utilization, and workflow efficiency. The study hypothesizes that remote radiotherapy serves as an efficient, cost-effective alternative to conventional planning methods, with the potential to reduce treatment backlogs, enhance throughput, and improve care quality. The study provides critical insights into the feasibility of remote planning in LMIC, with implications for informing future strategies to scale radiotherapy access in resource-constrained settings through digital innovation and clinical workflow optimization.

## Methods

### Study Design and Sample

A prospective, observational, descriptive study aimed at assessing the feasibility and efficacy of remote radiotherapy treatment planning feasibility will be conducted at CMJAH in Johannesburg, South Africa. The primary focus is on assessing implementation feasibility and workflow impact, rather than achieving statistical significance; no formal power calculations were performed prior to study initiation.

The pilot study aims to enroll 100 adult patients with histologically confirmed cervical, breast, prostate, head and neck, or rectal cancer. This sample size is deemed sufficient to assess operational efficiency feasibility and system integration and to generate preliminary data regarding the clinical usefulness and workflow efficiency of remote treatment in a resource-constrained environment.

### Study Settings

CMJAH hosts the busiest radiation oncology unit in South Africa, receiving the highest number of national radiotherapy referrals annually. However, the unit faces significant treatment delays due to severe equipment and human resource constraints. Reported wait times range from 12 months to 18 months for breast cancer and extend up to 5 years for prostate cancer [28].

Evidence from clinical trials demonstrates that delayed initiation of radiotherapy is associated with increased risk of local recurrence across several tumor types in cancer patients [29]. The current patient workflow for treatment planning is shown in Figures S1 and S2 in Multimedia Appendix 1. This pilot study seeks to assess the integration of remote treatment planning to improve efficiency, standardization, and equitable access to quality care objectives aligned with broader global health equity efforts in oncology.

### Eligibility Criteria

#### Inclusion and Exclusion Criteria

To be eligible for participation in this pilot study, patients must meet all the following inclusion criteria: age ≥18 years; histologically confirmed diagnosis of cervical, breast, prostate, head and neck, or rectal cancer; presentation to the Radiation Oncology Division for curative radiotherapy; and provision of written informed consent.

Patients meeting these criteria are informed about the study in a private room, with a trained member of the research team. On-site translators are available to assist where necessary. For interested patients, the consent form will be explained in detail. Upon agreement, patients will provide written consent using documentation approved by the institutional Human Research Ethics Committee.

Exclusion criteria include patients younger than 18 years old, previous history of radiotherapy, and current need for emergency or palliative treatment.

#### Withdrawal of Participants

Participants have the right to withdraw from the study at any time, without penalty or effect on their ongoing clinical care. Researchers may exclude participants requiring urgent interventions, and those who discontinue participation prior to completing the required study procedures will be censored and excluded from analyses assessing the impact of remote planning on treatment timelines and workflow metrics.

### Recruitment Procedures

**Figure 1:**
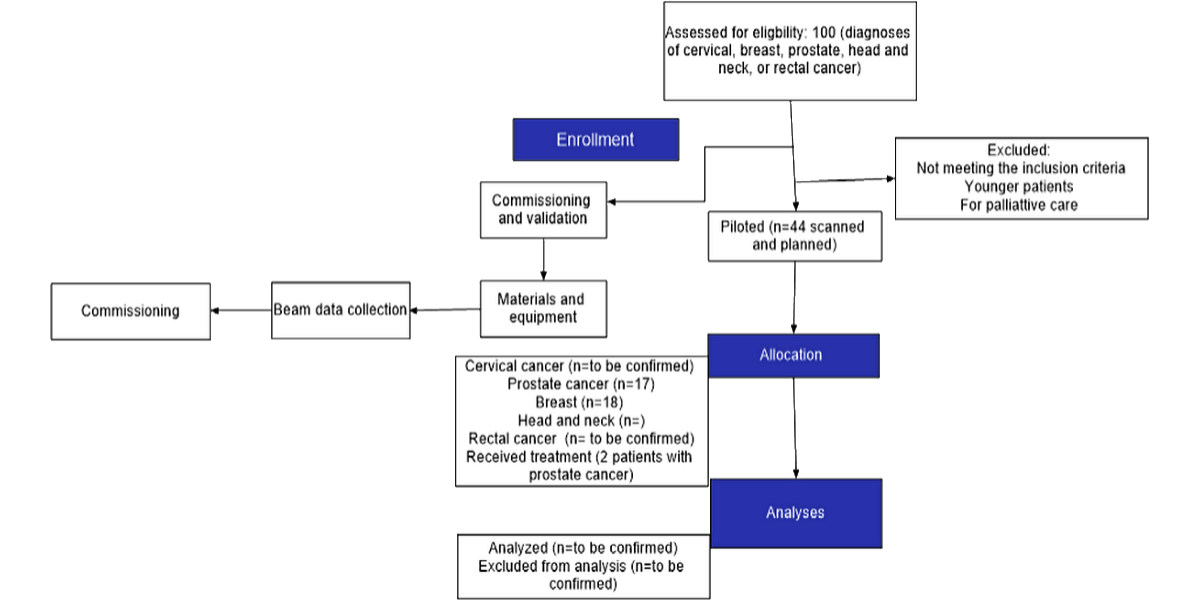
CONSORT (Consolidated Standards of Reporting Trials) flowchart of the remote radiotherapy treatment planning service.

Recruitment will be conducted in the Radiation Oncology Division at CMJAH. The recruitment and screening process is summarized in a simplified diagram (Figure 1) based on the CONSORT (Consolidated Standards of Reporting Trials) diagram, as described by Ruslin et al [30]. The study aims to enroll up to 100 patients in total; however, as of July 2024, 44 patients had been enrolled. The anticipated final cohort will include approximately equal representation across 4 cancer types, including cervical, breast, prostate, head and neck, and rectal cancers (approximately 25 per group), though final distributions may vary based on real-time patient presentation and eligibility. This sample size is deemed sufficient to evaluate feasibility and generate preliminary efficacy data on remote radiotherapy treatment planning services.

**Figure 2:**
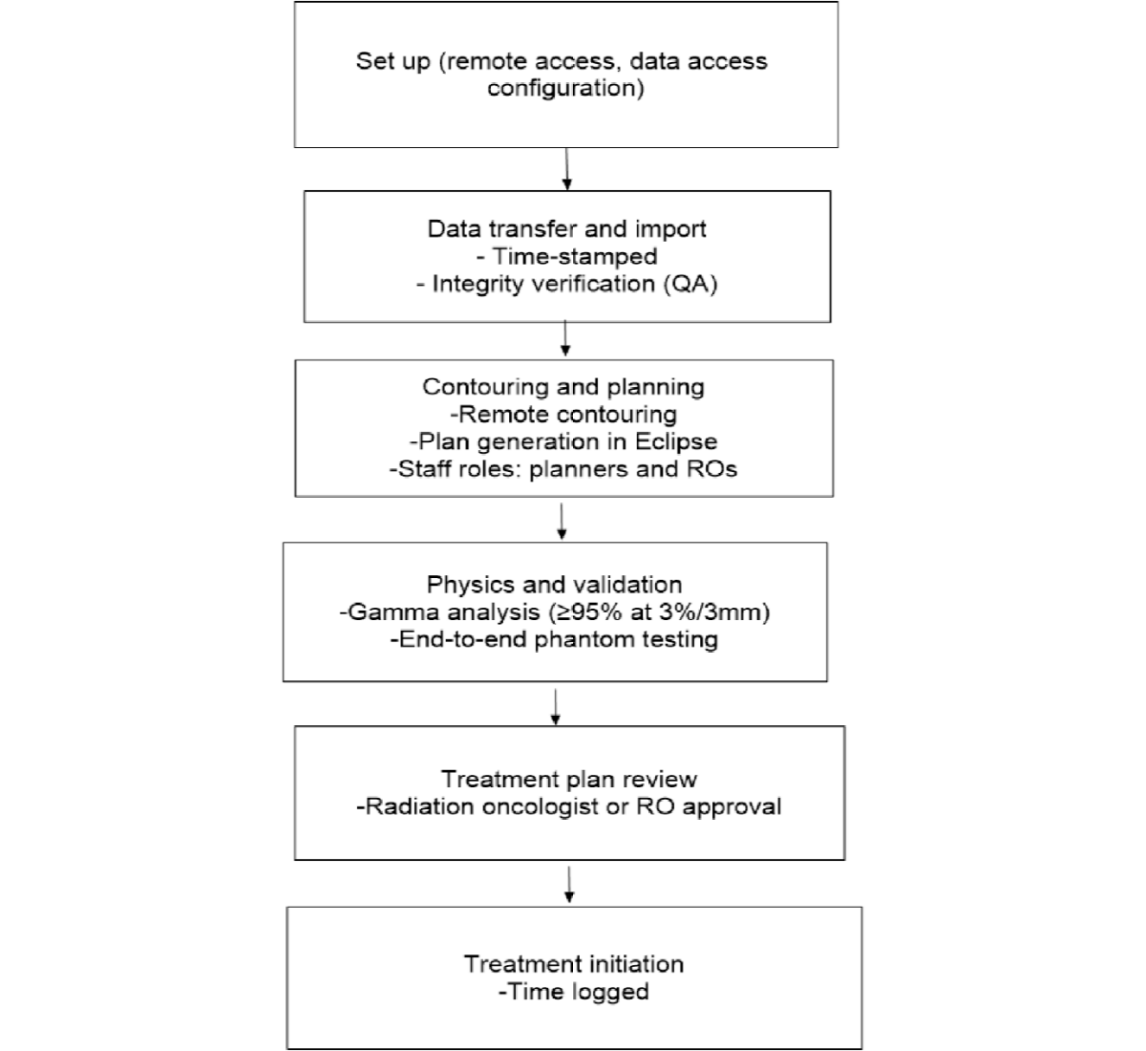
Critical decision points and corresponding time stamps are meticulously recorded at each stage of the process. QA: quality assurance; RO: radiation oncologist.

To identify eligible participants, all individuals presenting to the Radiation Oncology Division at CMJAH will be screened by the clinical staff following initial consultation and staging (as illustrated in Figure 2).

Prior to enrollment, participants will receive comprehensive information regarding the risks and potential implications of study participation. Informed consent will be obtained after confirming the participant’s understanding and agreement to participate in the study

### Feasibility and Efficacy Assessment

To evaluate the potential for integrating remote treatment planning within resource-constrained clinical settings, the study was designed in 2 phases: Phase 1 (feasibility assessment) and Phase 2 (efficacy assessment). This study focuses solely on Phase 1, which evaluates the operational and technical feasibility of remote radiotherapy planning. Phase 2, which will assess clinical efficacy outcomes, is planned for future research.

#### Phase 1: Feasibility Assessment

The feasibility of remote radiotherapy planning was evaluated through strategic modifications to the existing radiotherapy workflow to optimize task execution, enhance data interoperability, and streamline coordination among clinical teams. The components and performance metrics described in the following sections were applied (see Figure 2).

#### Assessment of Workflow Integration Feasibility

The integration of remote treatment into existing operations was assessed by documenting the time metrics for core planning activities, including remote contouring, treatment plan generation, and review of plans by radiation oncologists. These data will serve to describe the operational characteristics inherent to the remote planning process.

#### System Integration and Data Integrity

The assessment of system integration and interoperability will involve comprehensive end-to-end testing of the Varian Eclipse TPS with Elekta Versa HD LINACs. This methodical process was designed to ensure seamless integration, reliable data transfer, and technical preparedness for clinical implementation.

#### Beam Characterization and Integration Validation

Validation of the integration of the remote TPS will be conducted through a comprehensive series of technical assessments. Consequently, qualified medical physicists conducted the comprehensive beam modeling validation, which included parameters such as percent depth dose (PDD) curves, beam profiles, and output factors (OFs), with uniformity verified across all 4 LINACs. Additionally, the calibration of computed tomography (CT)–to-electron density calibration curves will be rigorously validated to ensure dosimetric accuracy in patient simulations.

#### Dosimetric Analysis and Workflow Timing and Logging

The evaluation of dosimetric accuracy will be conducted using gamma index analysis, adhering to a predetermined acceptance threshold of ≥95% at 3%/3 mm criteria, thereby ensuring the clinical safety of remotely generated plans through meticulous timing and logging. Furthermore, time-stamped logs will be used to assess the duration of critical integration processes, encompassing data transfer setup, verification of CT-to-electron density calibration, treatment plan import and export, and QA system configuration.

#### Operational Readiness and Success Criteria

Feasibility success was defined by (1) accurate and uninterrupted data exchange between the TPS and treatment delivery system, (2) plan acceptability based on dosimetric QA, and (3) uninterrupted clinical operations without the need for manual overrides or unplanned troubleshooting.

#### Clinical Review and QA Documents

The review and approval of the clinical suitability of autocontours and final treatment plans will be conducted by study radiation oncologists. As such, all validation procedures, comprising beam and imaging data verification, dose calculations, and QA results, will be meticulously documented using standardized forms and institutional standard operating procedures, in accordance with international guidelines (eg, American Association of Physicists in Medicine [AAPM], IAEA).

#### Training Staff (Future Work)

Systematic training sessions on the TPaaS will be implemented to ensure consistent data collection and workflow adherence. The training will be conducted by a collaborative team that includes personnel from Siemens-Varian alongside the study investigators. The training curriculum will encompass user manuals, live demonstrations, video tutorials, and hands-on workshops meticulously designed for the TPaaS and Eclipse remote planning systems. The 3-day program will encompass methodologies for data collection, strategic planning of workflows, protocols for system access, and processes for ensuring QA. Staff competency will be evaluated through supervised trial runs, with follow-up on-site observation for 1 week to 2 weeks to ensure sustained workflow integration. All training activities will be executed in accordance with ethical standards approved by the institutional review board.

#### Summary of Phase 1

In summary, successful integration of remote treatment planning will be defined by (1) technical interoperability-reliable transmission of imaging and planning data between LINAC systems; (2) dosimetric reliability and clinical acceptability of remotely generated images confirmed through standardized thresholds, as verified through patient-specific QA metrics; and (3) workflow sustainability, or the demonstrated ability to implement the remote planning workflow within existing clinical operations, without unplanned interruptions or manual interventions.

#### Phase 2 Efficacy Assessment (Future Work)

In contrast to the technical feasibility focus of Phase 1, Phase 2 will assess the operational effectiveness of the integrated remote planning system for improving clinical workflow efficiency and reducing treatment delays. The operational metrics described in the following paragraph will be evaluated.

For comparative workflow analysis, the impact of remote planning will be evaluated at key workflow stages, including the time from initial patient presentation to scanning, time from scanning to contour approval, time from contour approval to plan acceptance, and total time from patient presentation to treatment initiation. In addition, QA will be timed and quantitatively compared with historical data or matched in-person control cohorts using a 1:1 matching methodology. The primary outcome is reduction in total time to treatment; secondary outcomes include increased patient throughput and reductions in backlogs.

Although radiotherapy planning is hypothesized to reduce delays, its effectiveness for accelerating treatment workflows in resource-constrained settings will be rigorously evaluated. This phase aims to determine whether a remote planning system provides measurable operational benefits in a routine clinical environment.

A detailed analysis of Phase 2, including its methodology and findings, will be presented in future studies.

### Proposed Workflow: Remote Radiotherapy Planning

This study uses the Eclipse (Varian) remote radiotherapy planning system, integrated AI-supported tools, and institutionally standardized QA processes. The system adheres to Digital Imaging and Communications in Medicine (DICOM) standards and is designed to facilitate efficient, high-quality treatment planning across distributed teams. Radiation oncologists and dosimetrists can create customized plans, visualize imaging data, and collaborate remotely. Varian’s systems ensure the accuracy and safety of patient data.

CT is performed at the CMJAH Radiation Oncology Division. Once acquired, CT data sets are uploaded to a secure server hosted within the facility and accessed remotely via a secure virtual private network connection installed by Siemens-Varian Healthineers in compliance with the South Africa Protection of Personal Information Act.

**Figure 3:**
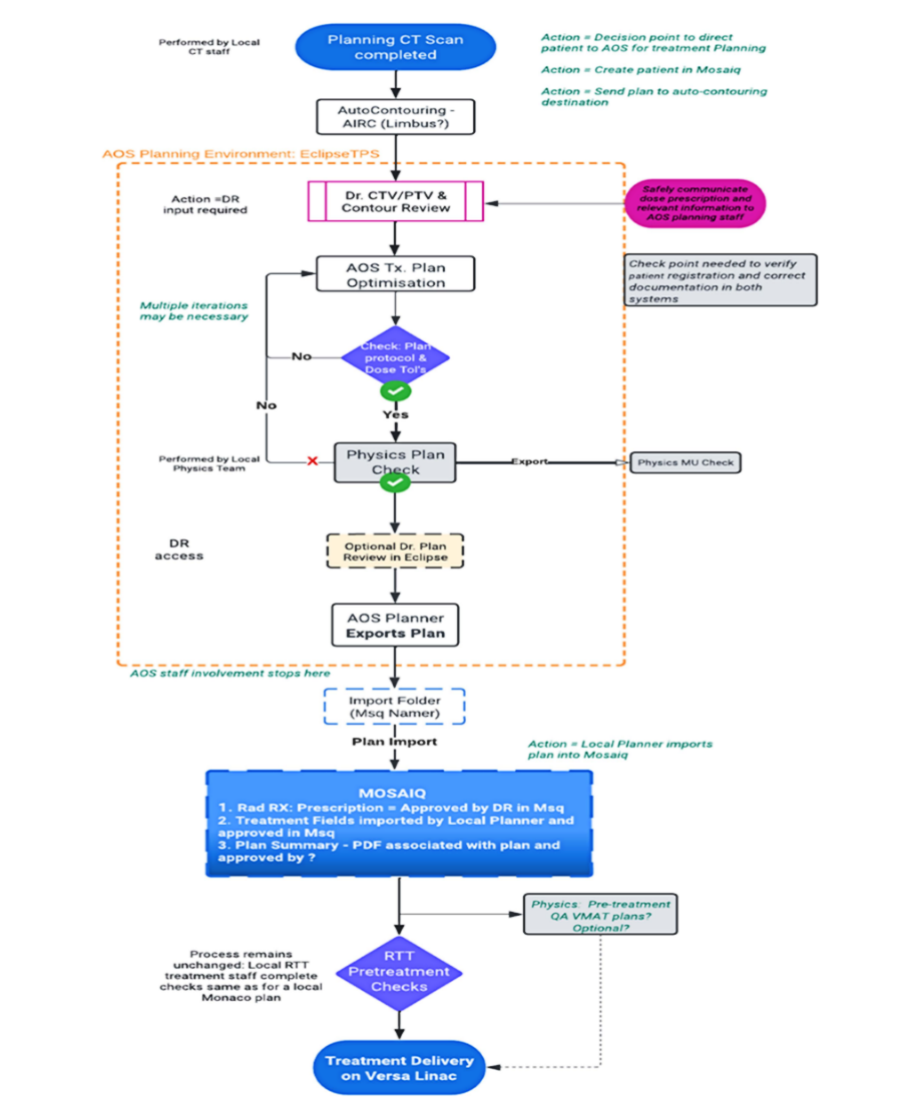
Remote radiotherapy planning steps. AOS: advanced oncology solutions; AIRC: artificial intelligence companion radiation-based radiotherapy; CT: computed tomography; CTV: clinical target volume; DR: dose rate; LINAC: linear accelerator; Msq: Mosaiq; MU: monitor unit; PTV: planning target volume; QA: quality assurance; TPS: treatment planning system; ToI: tolerance; Tx: radiation treatment or treatment plan; RTT: radiation therapist; VMAT: volumetric modular arc therapy.

Organs at risk (OARs) are delineated using the Monaco and Limbus AI auto-contouring systems, as illustrated in Figure 3. These contours are then reviewed and adjusted by an on-site radiation oncologist as needed to ensure anatomical fidelity and protocol adherence.

Remote planners located in the United States and India access patient data sets and proceed to develop treatment plans within the Eclipse TPS. AI-assisted optimization tools facilitate plan generation, which is manually verified by dosimetrists before submission for approval.

Collaborative review between radiation oncologists and remote planners is facilitated through encrypted platforms (ie, video calls and secure messaging) for plan finalization. Upon approval, the plans are digitally signed off by the radiation oncologist and transferred for QA in accordance with established clinical protocols.

Following plan generation, medical physicists at CMJAH conduct rigorous beam modeling verification and patient-specific QA checks using the Octavius 4D phantom system. This step confirms dosimetric accuracy and equipment compatibility.

The radiotherapists subsequently verify the preparedness for treatment, schedule sessions, and perform final verification steps. The in-house Monaco plan is used in parallel to safeguard the clinical integrity.

The delineation of roles and responsibilities across clinical staff (radiation oncologists, medical physicists, radiotherapists, and remote planners) is illustrated in Figure 3. This methodical, phased approach ensures patient safety, data confidentiality, and the precision of clinical outcomes during remote planning.

Any implementation of remote planning requires integrating a comprehensive review step without compromising clinical standards. Future studies will focus on incorporating automated review support tools or scheduling adjustments to reduce delays while maintaining rigorous QA protocols, enhanced scheduling algorithms to minimize idle time between planning and treatment, and implementation of remote plan review dashboards for real-time tracking.

This structured, multitiered approach is expected to improve workflow efficiency, preserve safety standards, and provide a scalable model for radiotherapy services in LMIC.

### Data Collection

Table 1 shows how the assessments will be conducted.

In this study, a standardized data capture sheet (Multimedia Appendix 2) will be used to collect each participant’s clinical and procedural data. Variable recorded will include sociodemographics (age, sex); cancer type; treatment details; treatment planning type; QA metrics; and key dates, including first consultation, CT simulation, treatment planning initiation, treatment plan approval, and treatment completion. Although patient demographics are not directly linked to the primary aims of this study, they will be collected to contextualize the representativeness of the cohort and support post hoc analyses examining whether characteristics such as age, sex, or disease-specific characteristics influence workflow efficiency or waiting times. This ensures transparency and enhances the generalizability of findings across diverse patient populations. Including this information allows for a more comprehensive understanding of the clinical applicability and scalability of remote planning systems across diverse patient populations.

**Table 1:** Assessment of the pilot.

Items	Consent	Protocol	Diagnosis	Volumes	Contouring	Treatment
CT^a^ scans	✓	—^b^	—	—	—	—
Cervical	—	✓	✓	✓	✓	✓
Head and neck	—	✓	✓	✓	✓	✓
Rectal	—	✓	✓	✓	✓	✓
Breast	—	✓	✓	—	—	✓
Prostate	—	✓	✓	✓	✓	✓

^a^CT: computed tomography.

^b^Not applicable.

### Data Collection on Commissioning and Equipment

This study aims to evaluate the feasibility and operational efficiency of remote treatment planning in resource-constrained settings, specifically for accurate delineation of gross tumor volumes and OARs. Key commission activities include the acquisition and verification of the CT-to-electron density curve, an essential component for ensuring accurate dose calculations. Additional details on the remote planning process are provided in Multimedia Appendix 3.

The commissioning will encompass the integration of 4 Elekta Versa HD LINACs with the Varian Eclipse TPS, supported by a comprehensive beam-matching process to standardize data across machines, enhancing flexibility and accuracy. Key measurements, including PDD curves, profiles, and OFs, will be acquired using PTW’s BEAMSCAN water tank in accordance with the AAPM and IAEA guidelines. Focused beam validation will center on photon and electron energies of 6X, 10X, and 6X flattening filter-free photons suitable for Eclipse modeling [31]. Validation of all measurements will be carried out by qualified medical physicists. This includes beam data verification (PDD curves, beam profiles, OFs), end-to-end phantom testing for dose accuracy, patient-specific QA using gamma index analysis (≥95% pass at 3%/3 mm), and review of CT-to-electron density calibration for dose calculation reliability.

Multileaf collimator (MLC) characteristics, including dosimetric leaf gap, and transmission factors will be calibrated using a sweeping-gap technique, with iterative refinements to ensure accuracy across all energy levels [32].

Comprehensive system validation will follow the IAEA’s end-to-end methodology, simulating clinical scenarios using phantoms to test dose distribution integrity across various field sizes and geometries [31]. Fine-tuning will continue through QA, including validation using gamma analysis for IMRT/VMAT plans, aiming for a minimum 95% pass rate at 3%/3 mm criteria [32].

The final QA process will confirm the technical readiness for clinical implementation across all 4 LINACs, ensuring reproducibility and dose accuracy for patient treatment under remote planning paradigms.

### Quality Control

Prior to pilot study commencement, the research team will receive structured training on TPaaS, QA protocols, administration procedures, and clinical documentation. During the study, attending radiation oncologists will oversee prescription validation for each cancer type. Following CT simulation, imaging data will be securely backed up via Citrix systems, as depicted in the workflow in Figure 2, with audit trails in place for monitoring data access and modification. Reasons for any participant discontinuing treatment will be recorded.

### Statistical Analysis

Sociodemographic, clinical, and dosimetric parameters, along with CT images of patients, will be extracted and anonymized, entered into a REDCap (Research Electronic Data Capture) database, followed by exportation into STATA software (v.19) for statistical analysis. Descriptive statistics will summarize patient characteristics, cancer type, and planning timelines. Associations between categorical variables (planning type and treatment delays) will be examined using chi-square and Fisher exact tests [33], and odds ratios and CIs will be calculated. For continuous outcomes (eg, time intervals), *t* tests or nonparametric equivalents will be used where appropriate. Multivariable logistic regression analysis will be considered to adjust for potential confounders (eg, age, sex, cancer type, treatment intent) in outcomes such as plan approval delay or planning method efficacy. Statistical significance will be set at *P*<.05.

### Ethical Considerations

This pilot study was approved by the Human Research Ethics Committee (Medical) of the University of Witwatersrand, Johannesburg (certificate number M231076). All participants will provide written informed consent before enrollment.

Participants will receive full information regarding the study procedures, data collection, and voluntary nature of participation before signing the consent form. They are explicitly informed of their right to withdraw at any time without facing adverse consequences. No monetary compensation will be provided; however, participants will benefit from free access to treatment and diagnostic services directly related to the pilot study.

Strict privacy protocols ensure that all medical data are exclusively stored on the CMJAH server, with encryption protocols and unique study codes to maintain confidentiality. No participant data will be transferred to or retained on external storage systems. Siemens-Varian will fund the hybrid remote and AI method for 100 patients at approximately R3000 (US $170.51) per plan (total cost R300,000 [US $17,050.60]). Radiation therapy costs will be covered under the Radiation Oncology Syndicate at Wits Health Consortium covering the associated logistical costs such as patient transportation.

## Results

The study, which began in November 2023, is currently in the enrollment phase. As of July 2024, more than 44 patients were recruited for radiotherapy planning. Among these 44 patients, 17 (39%) are undergoing planning for prostate cancer, 18 (41%) for breast cancer, and 9 (21%) for head and neck cancer. The project was funded in May 2023, with data collection projected to end in May 2026.

The commissioning and validation of the Varian Eclipse TPS integrated with Elekta Versa HD LINACs involved rigorous assessments to ensure accurate beam modeling and dosimetric reliability. Measurements included profiling PDD curves and OFs across relevant photon energies. The process also entailed CT-to-electron density curve verification, optimization of MLC transmission characteristics, and dose calculations using the anisotropic analytical algorithm. 

The validation procedure was led by a team of qualified medical physics and clinical staff. Preliminary results, as summarized in Tables 2 and 3, indicate that transmission of factors of the MLC consistently remained <0.5% across all energy levels, exhibiting a variation ranging from 0.3% to 0.5%, which aligns with the findings of previous studies [32,34] and are significantly well below the 2% threshold set forth by the AAPM Task Group No 50 [35]. The data confirm the LINAC calibration integrity, with dose deviations maintained within clinically acceptable thresholds.

**Table 2:** Measured dosimetric leaf gap (DLG) and multileaf collimator (MLC) transmission.

Energy level	DLG (mm)	MLC transmission, %
6X	0.31	0.44
10X	0.47	0.45
6X flattening filter free (FFF)	0.38	0.29

**Table 3:** Reference dosimetry results using an independent chamber.

Energy level	Expected dose (Gy)	Measured dose (Gy)	Difference
6X	1.000	1.0018	–0.18
10X	1.000	1.0164	–1.64
6X flattening filter free (FFF)	1.000	1.02598	–2.60

Minor discrepancies were observed between measured and modeled data, particularly for small field sizes and shallow depths, which were attributed to beam hardening and electron contamination. The measurement results, as outlined in Table 3, validated that the LINACs were accurately calibrated to deliver the correct specified doses. The measured doses closely matched the expected values, exhibiting deviations that remained within acceptable limits, thereby ensuring the accuracy and reliability of the LINAC systems in clinical settings.

Dosimetric validation was performed using QA testing with phantom models and cross-calibration, meeting IAEA standards and achieving dose deviations within acceptable limits. Remote infrastructure testing using mock patient data sets demonstrated technical readiness for clinical application, with gamma index pass rates exceeding 90% (see Table 4).

Detailed results will be included as part of a comprehensive analysis within the complete manuscript, offering an in-depth overview of system performance, workflow efficiency, and preparedness for clinical integration. Preliminary results are anticipated to be published by mid-2025 and disseminated through high-impact peer-reviewed journals (quartile 1 and quartile 2), reports, conference proceedings, and other relevant platforms, providing robust insights and potential recommendations based on the study’s outcomes.

**Table 4:** Patient-specific quality assurance results for the 5 mock patients with distance to agreement (DTA) of 3 mm and dose difference (DD) of 5%.

Patient	Location	Results, %
VarianTest 1	Prostate	98.90
VarianTest 2	Head and neck	91.90
VarianTest 3	Breast	97.50
VarianTest 4	Prostate	99.20
VarianTest 5	Breast	98.80

## Discussion

### Key Findings and Interpretation

This study hypothesizes that remote radiotherapy planning can reduce time delays, reduce associated costs, and improve planning efficiency and patient care delivery in resource-constrained settings. As a preliminary evaluation of feasibility and efficacy of remote radiotherapy planning, this study explores an off-site planning service, as an innovative approach to addressing longstanding infrastructural and workforce challenges in radiation oncology in LMIC.

At the time of this study’s protocol development, the Radiation Oncology Division at CMJAH experienced considerable diagnostics delays due to limited imaging equipment and increased patient volumes. For example, waiting time for magnetic resonance imaging scans can extend up to 7 months, and CT services were frequently disrupted by technical failures. These delays highlight systemic barriers prevalent across Gauteng public hospitals, where resource constraints and high patient-to-equipment ratios hinder timely cancer care.

Conventional radiotherapy workflows at CMJAH, detailed in Figure S2 in Multimedia Appendix 1 and Multimedia Appendix 4 [36,37], often rely heavily on in-person methods to generate plans. In recent years, off-site treatment services in radiation oncology have gained traction [38], enabling planning expertise to be provided remotely, even across countries [39,40]. In settings like Canada, the United States, the United Kingdom, and Portugal, these approaches have been driven by concerns over SARS-CoV-2 transmission during the COVID-19 pandemic [41], which was associated with heightened mortality rates. These models allowed for interregional and international redistribution of planning expertise and fewer treatment disruptions [42].

Despite this, limited data exist on the performance and scalability of off-site radiotherapy planning services in radiation oncology in LMIC, particularly in SSA [27], where infrastructural limitations and concerns about patient privacy remain significant barriers [43]. Standardization of clinical studies is essential to evaluate the feasibility and quality of these services, especially in resource-constrained settings. Early evidence from remote planning services, such as with Monaco or Eclipse systems, suggests these platforms can potentially reduce backlogs, optimize resources, and maintain plans [44,45]. Previous commissioning studies have shown these services can alleviate treatment delays and improve radiotherapy delivery [45], supporting their investigation in this study. This underscores the need for structured evaluation in LMIC settings, such as CMJAH.

Unfortunately, implementation of remote radiotherapy planning services at CMJAH presented challenges. These included short trial license periods, treatment couch discrepancies requiring re-planning, and the retraining of staff accustomed to in-person systems like Monaco. Technical limitations, such as restricted access for medical physicists, necessitated collaborative workarounds involving therapists and planners. Workflow integration with TPaaS required effective communication among internal and external teams (eg, doctors, medical physicists). Additionally, therapists managed printing, exporting, and preparing treatment data due to limited access to the Eclipse system for medical physicists.

In this study, we anticipate that remote radiotherapy planning services will effectively support treatment planning, as observed in previous studies conducted during the COVID-19 pandemic [41]. Drawing on lessons from the COVID-19 pandemic and remote planning models in HIC, this study aims to quantify parameters and generate insights into the feasibility and early efficacy of the remote planning services, both scientifically and clinically. These findings will inform cancer treatment planning, particularly in resource-constrained settings, and contribute to the broader discourse on modernizing cancer care in LMIC through technology-enabled solutions.

Although remote planning offers a promising strategy to reduce wait times and workflow efficiency, successful implementation demands mitigation of specific technical and systemic barriers. First, reliable and secure data transfer systems are critical to prevent workflow delays and protect patient privacy. Network connectivity and data security protocols must be robust to support real-time collaboration between remote and on-site teams. Second, interoperability between planning systems and software tools must be optimized to ensure consistency in target delineation and dose delivery. Third, effective communication and coordination are essential to streamline decision-making and avoid process bottlenecks, especially when real-time consultation is required.

Finally, training demands must be addressed through structured educational programs to facilitate clinician adaptation to remote workflows. Addressing these challenges requires careful workflow optimization, investment in technology infrastructure, and comprehensive training programs to maintain efficiency and ensure the quality and safety of patient care.

Implementing remote planning to reduce waiting times requires specific workflow changes, which introduce potential barriers that must be addressed to ensure feasibility and efficacy. Key workflow modifications include the introduction of secure remote access for data transfer, ensuring seamless integration between remote and local planning systems, and scheduling adjustments to facilitate real-time communication among team members. However, these changes present challenges, such as the need for robust data security measures to maintain patient confidentiality, potential delays due to network connectivity issues, and variations in the performance of different contouring and planning software tools. Additionally, effective communication between remote and on-site staff is critical to avoid workflow disruptions.

To overcome these barriers, investments in secure, high-speed data transfer infrastructure, standardized software compatibility protocols, and comprehensive team training are required. Furthermore, optimizing communication workflows through scheduled review sessions and clearly defined roles can mitigate delays and enhance process coordination. Addressing these challenges is essential to fully realize the benefits of remote planning in reducing waiting times without compromising treatment quality and safety.

Moreover, training demands must be addressed through structured educational programs to facilitate clinician adaptation to remote workflows. Technological variability, internet connectivity limitations, and software licensing constraints can introduce delays, offsetting potential time savings. For instance, routine synchronization of imaging and planning data can add an estimated 5 minutes to 15 minutes per case, depending on case complexity and network bandwidth.

To enhance system readiness, this study implemented proactive measures, including predefined data transfer templates, optimized scheduling, and system configuration protocols. These efforts aim to reduce integration-related delays and ensure that remote planning can be conducted without compromising clinical safety, data security, or overall treatment timelines.

### Limitations

This study has several limitations. First, as a single-site pilot study, generalizability may be restricted, and findings may not be immediately transferable to other LMIC or health systems. However, the results provide a critical groundwork for evaluating remote radiotherapy workflows in resource-limited contexts. The adapted data collection tools, although based on validated instruments, require further contextual validation. Additionally, real-time staff engagement data and cross-platform integration (eg, Eclipse and Mosaiq) challenges require further study to support optimization and scale-up. Second, although the data sheet was adapted from validated questionnaires, it has not yet undergone extensive validation within the context of this study. However, this pilot will inform its final version.

Although the assessment of the feasibility and scalability of this model in various LMIC constitutes a primary objective, this study does not incorporate a formal cost analysis. The financial implications associated with integration and implementation are currently ambiguous, potentially constraining the generalizability of findings to other resource-limited environments.

Technical barriers including short-term licensing, planning system discrepancies, and limited access for physicists impacted implementation. Furthermore, the limited study duration may not fully capture longitudinal outcomes such as plan durability or patient satisfaction. Despite these limitations, this is the first study in SSA evaluating the feasibility of a remote (AI and hybrid remote) radiotherapy planning model, which could offer a rare opportunity to document early insights into hybrid and AI-supported planning models in SSA to enhance cancer treatment efficiency, reduce wait times, and improve the patient experience.

### Future Directions

Future research should explore AI-generated contouring, plan quality, time efficiency, staff and patient satisfaction, and privacy concerns and incorporate detailed cost-effectiveness analyses and implementation frameworks to assess the economic feasibility, scalability, and sustainability of the model within various health care systems in LMIC. Data collection on staff perspectives and any workflow adjustments observed during the integration process is essential to optimize remote radiotherapy planning. Research should include evaluating workflow adjustments, interoperability challenges, and clinical efficiency during integration of the Eclipse and Mosaiq systems. Such studies should also explore staff experiences and technical requirements to optimize integration, offering valuable insights for broader implementation across similar health care settings. Enhanced understanding of these integration requirements would support improved planning and resource allocation, ultimately contributing to streamlined radiotherapy processes and patient care. Comparative studies of Monaco and Scripting planning systems are needed to evaluate OAR sparing, inverse planning, and efficiency. Insights from these studies will support the broader adoption of remote radiotherapy, optimize workflows, and enhance patient care.

### Conclusion

This study introduces and evaluates the feasibility and initial efficacy of remote radiotherapy planning as a tool to improve treatment planning and a solution for workflow inefficiencies in oncology care in LMIC. Targeting high-burden malignancies such as cervical, prostate, breast, head and neck, and rectal cancers, this approach holds the potential to optimize resource use, streamline clinical operations, and enhance care delivery. By leveraging remote infrastructure and international expertise in such environments, remote planning may bridge the gaps in oncology care and improve cancer care delivery where resources are limited. The findings have significant implications for global health care, particularly in advancing equitable access to high-quality radiotherapy services in underserved regions.

## Appendix

Multimedia Appendix 1 Patient workflows.

Multimedia Appendix 2 Data capture in the Radiation Oncology Department, Charlotte Maxeke Johannesburg Academic Hospital (CMJAH).

Multimedia Appendix 3 Remote planning process.

Multimedia Appendix 4 Conventional radiotherapy workflows at Charlotte Maxeke Johannesburg Academic Hospital (CMJAH).

## Abbreviations

AAPM: American Association of Physicists in Medicine
AI: artificial intelligence
CMJAH: Charlotte Maxeke Johannesburg Academic Hospital
CONSORT: Consolidated Standards of Reporting Trials
CT: computed tomography
DICOM: Digital Imaging and Communications in Medicine
HIC: high-income countries
IMRT: intensity-modulated radiotherapy
LINAC: linear accelerator
LMIC: low and middle-income countries
MLC: multileaf collimator
OAR: organ at risk
OF: output factor
OR: odds ratio
PDD: percent depth dose
QA: quality assurance
REDCap: Research Electronic Data Capture
SSA: sub-Saharan Africa
TPaaS: treatment planning as a service
TPS: treatment planning system
VMAT: volumetric-modulated arc therapy
WHO: World Health Organization

## Data Availability

Data from this pilot study will be available from the corresponding author upon reasonable request. Patient data will be deidentified before any sharing requests.
