# Supplementary material for: Implementing Remote Radiotherapy Planning to Increase Patient Flow at a Johannesburg Academic Hospital, South Africa: Protocol for a Prospective Feasibility Study
Source: JMIR Res Protoc. 2025 Jul 28;14:e60131. doi: 10.2196/60131 (PMC12340459; doi:10.2196/60131)
Supplement: Multimedia Appendix 1 [file resprot_v14i1e60131_app1.docx]

**Figure S1**. Current workflow through the CMJAH Radiation Oncology Department as illustrated in the patient information pamphlet.


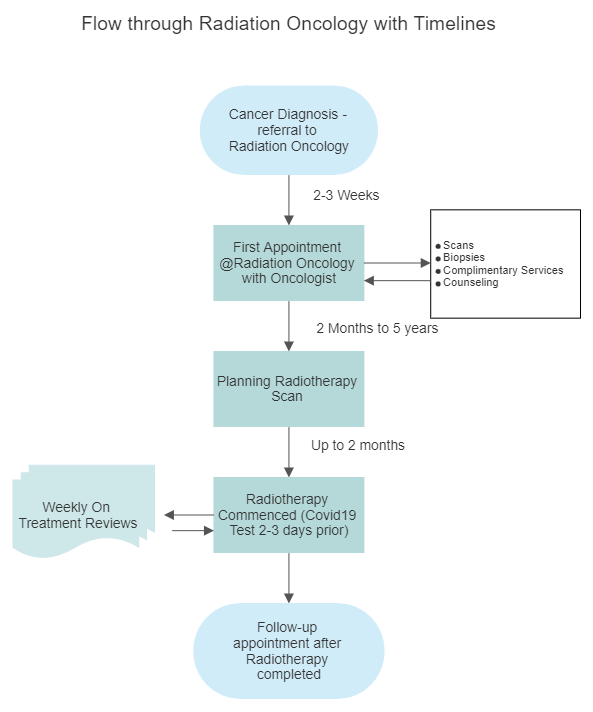


**Figure S2.** Typical workflow implemented in the radiation oncology department.

Planning CT Scan

Contouring

Planning

Assessment of plan and approval

Quality assurance
