# Supplementary material for: Implementing Remote Radiotherapy Planning to Increase Patient Flow at a Johannesburg Academic Hospital, South Africa: Protocol for a Prospective Feasibility Study
Source: JMIR Res Protoc. 2025 Jul 28;14:e60131. doi: 10.2196/60131 (PMC12340459; doi:10.2196/60131)
Supplement: Multimedia Appendix 2 [file resprot_v14i1e60131_app2.docx]

| Category | Description |
| --- | --- |
| Demographics | Age, gender |
| Cancer characteristics | Type of cancer, stage at diagnosis |
| Treatment | The total dose of radiation, fractionation, dose per  Fraction, technique, boost |
| Type of treatment planning | Method of radiation therapy planning (Routine  Departmental planning or hybrid remote/AI) |
| Quality assurance | Number of plans that meet the target volume constraint,  Number of plans that meet the OARs constraints |
| Dates | Date of the first consultation, Date of CT simulation  Date of initiation and completion of treatment planning |
|  | Date of approval of treatment planning  Date of initiation and completion of the quality assurance of the planned treatment  Date of initiation and completion of treatment |

Table S1. Data capture at the Radiation Oncology, CMJAH
