# Supplementary material for: Implementing Remote Radiotherapy Planning to Increase Patient Flow at a Johannesburg Academic Hospital, South Africa: Protocol for a Prospective Feasibility Study
Source: JMIR Res Protoc. 2025 Jul 28;14:e60131. doi: 10.2196/60131 (PMC12340459; doi:10.2196/60131)
Supplement: Multimedia Appendix 3 [file resprot_v14i1e60131_app3.docx]

**Remote therapy planning**

Remote radiotherapy planning represents a transformative approach to modern oncology care, with the potential to significantly reduce treatment backlogs and enhance care delivery. By allowing planning to be conducted at remote sites, this approach not only alleviates the pressure on overburdened facilities but also enhances access to specialized care. With careful consideration of the associated challenges, remote planning can be integrated into existing workflows to create a more efficient, scalable, and patient-centered radiotherapy service. [1]. The Radiation Planning Assistant (RPA) represents a significant advancement in the field of radiotherapy, offering a solution to the challenges of limited access to specialized radiotherapy planning expertise. By automating key components of the planning process, RPA not only enhances the accessibility and efficiency of radiotherapy planning but also ensures consistency and high quality in treatment plans. As a result, patients in remote or resource-limited settings can receive the same level of care traditionally available only in major cancer centers, leading to better outcomes and more equitable cancer care [2]. The RPA has proven its technical effectiveness through robust optimization and feasibility in generating RT plans for cervical, head and neck, and breast cancer. The software’s ability to consistently produce high-quality, clinically viable plans across these diverse and challenging cancer sites underscores its potential to revolutionize radiotherapy planning, making it more accessible and efficient, particularly in remote or resource-constrained environments. These successes affirm RPA's role in advancing the quality of cancer care through automated, yet highly effective, radiotherapy planning solutions [2-4].

Studies comparing RPA-generated plans to those created manually by experienced clinicians have generally shown that RPA can produce plans that are comparable in quality and accuracy. However, there are nuances depending on the specific aspect of the planning process being evaluated. A study has demonstrated that the use of a fully automated RPA for head and neck cancer patients can significantly reduce waiting times, addressing a critical challenge posed by staff shortages. By reducing waiting times and enabling the provision of advanced radiotherapy techniques, RPA technology not only enhances operational efficiency but also broadens access to high-quality cancer care. This development is particularly valuable for clinics with limited resources, as it allows them to offer treatments that were previously beyond their reach, thereby improving patient outcomes and equity in healthcare access [5].

In the case of head and neck cancers, the radiotherapy planning workflow is particularly complex due to the need for precise targeting of the tumor while sparring surrounding critical structures. The use of an automated system like RPA involves several key steps, with a focus on contouring the gross tumor volume and revising auto-generated organs at risk (OARs) and clinical target volumes (CTVs). However, the final refinement and approval by a clinician ensure that the plan meets the high standards required for effective and safe radiotherapy in this challenging anatomical region. This integrated approach leverages the strengths of both automation and human expertise, resulting in optimized treatment plans that can improve patient outcomes. [1]. A study investigating the use of a fully automatic treatment planning system of cervical cancer found that this approach is both effective and potentially a reliable option for low-resource constrained clinics [4]. This finding is particularly significant as it highlights the potential for automated systems to enhance the accessibility and quality of cancer care in settings where resources and specialized expertise are limited. The adoption of such systems could lead to significant improvements in the equity and efficiency of cancer care, ultimately enhancing patient outcomes in low-resource settings.

**References**

1. Olanrewaju A, Court LE, Zhang L, Naidoo K, Burger H, Dalvie S, Wetter J, Parkes J, Trauernicht CJ, McCarroll RE, Cardenas C, Peterson CB, Benson KRK, du Toit M, van Reenen R, Beadle BM. Clinical Acceptability of Automated Radiation Treatment Planning for Head and Neck Cancer Using the Radiation Planning Assistant. Practical Radiation Oncology 2021;11(3):177–184. PMID:33640315
2. McGinnis GJ, Ning MS, Beadle BM, Joubert N, Shaw W, Trauernich C, Simonds H, Grover S, Cardenas CE, Court LE, Smith GL. Barriers and Facilitators of Implementing Automated Radiotherapy Planning: A Multisite Survey of Low- and Middle-Income Country Radiation Oncology Providers. JCO Global Oncology 2022;(8):1–10. PMID:35537104
3. Kisling K, Zhang L, Simonds H, Fakie N, Yang J, McCarroll R, Balter P, Burger H, Bogler O, Howell R, Schmeler K, Mejia M, Jhingran A, Court L, Beadle BM. Fully automatic treatment planning for external-beam radiation therapy of locally advanced cervical cancer: A tool for low-resource clinics. Journal of Global Oncology 2019;2019(5):1–8. PMID:30629457
4. Court LE, Kisling K, McCarroll R, Zhang L, Yang J, Simonds H, du Toit M, Trauernicht C, Burger H, Parkes J, Mejia M, Bojador M, Balter P, Branco D, Steinmann A, Baltz G, Gay S, Anderson B, Cardenas C, Jhingran A, Shaitelman S, Bogler O, Schmeller K, Followill D, Howell R, Nelson C, Peterson C, Beadle B. Radiation planning assistant - A streamlined, fully automated radiotherapy treatment planning system. Journal of Visualized Experiments 2018;2018(134):1–9. PMID:29708544
5. Datta NR, Samiei M, Bodis S. Radiation therapy infrastructure and human resources in low- and middle-income countries: Present status and projections for 2020. International Journal of Radiation Oncology Biology Physics Elsevier Inc.; 2014;89(3):448–457. PMID:24751411
