# Supplementary material for: Implementing Remote Radiotherapy Planning to Increase Patient Flow at a Johannesburg Academic Hospital, South Africa: Protocol for a Prospective Feasibility Study
Source: JMIR Res Protoc. 2025 Jul 28;14:e60131. doi: 10.2196/60131 (PMC12340459; doi:10.2196/60131)
Supplement: Multimedia Appendix 4 [file resprot_v14i1e60131_app4.docx]

**Protocol**

**Assessing the Feasibility of Implementing Remote Radiotherapy Planning to Increase Patient Flow in Johannesburg Academic Hospital, South Africa: Protocol for a Prospective study**

Duvern Ramiah^1*^, Sonwabile Ngcezu^2^, Oluwatosin A. Ayeni^1^, Okechinyere Achilonu^3^, Mariam Adeleke^4^, Theo Nair^5^, Joseph Johannes Otten^2^, Daniel Mmereki^1*^

^1^University of the Witwatersrand, Faculty of Health Sciences, School of Clinical Medicine, Radiation Oncology, ^2^University of the Witwatersrand, Faculty of Health Sciences, School of Clinical Medicine, Medical Physics, Johannesburg, South Africa, ^3^University of the Witwatersrand, Faculty of Health Science, School of Public Health Epidemiology and Biostatistics Division, Johannesburg, South Africa, ^4^Department of Statistical Science, University of College London, London, United Kingdom, ^5^Siemens-Varian Healthineers, London, United Kingdom

^*^**Correspondence author**:

Duvern Ramiah MBCCh, MBA; Daniel Mmereki, PhD

Radiation Oncology, University of the Witwatersrand,

Charlotte Maxeke Johannesburg Academic Hospital, Radiation Oncology,

17 Jubilee Road, Area 348 Orange Block Level 4, Parktown, Johannesburg, 2193, South Africa

Phone: +27 (11) 488-4911

Email: [duvern.ramiah@wits.ac.za](mailto:duvern.ramiah@wits.ac.za); [daniel.mmereki@wits.ac.za](mailto:daniel.mmereki@wits.ac.za)

**This supplementary material includes four descriptive sections, and two figures.**

**Section A1: Current approach: patient flow through radiotherapy**

**Section A2: Typical workflow in a radiation oncology department**

**Section A3: Radiotherapy treatment process**

**Section A4: Remote therapy planning**

**Figure S1 Current workflow through the CMJAH Radiation Oncology Department as illustrated in the patient information pamphlet.**

**Figure S2 Typical workflow implemented in a radiation oncology department**

**Section A1:** **Current approach: patient flow through radiotherapy**

Patient flow in radiotherapy is a critical aspect of ensuring the timely [1] and safe initiation of cancer treatment. It involves a series of process maps that outline each step the patient undergoes, from initial examination room to final check-out. By mapping out each step of the process, radiation oncologists and healthcare teams can ensure that treatment is initiated safely and efficiently, ultimately leading to better patient outcomes. [2]. These process maps not only aid in reducing cycle time but also enhance the quality and safety of radiotherapy, making them a vital component of modern cancer care. Once the radiation oncologist examines a patient in the clinic and prescribes radiotherapy, the next step is to schedule a radiotherapy scan. This scan, often performed using CT, magnetic resonance imaging (MRI) positron emission tomography (PET), is critical in the treatment planning process. It allows for precise mapping of the tumor and surrounding tissues, ensuring that the radiation dose is accurately targeted to the malignancy while minimizing exposure to healthy tissue. The planning scan forms the foundation for the creation of a detailed, which includes determining the optimal angles, intensities, and durations of radiation delivery [3]. In the context CMJAH as the study setting, once this scan is completed, the patient is completed, the patient is informed that the next step is to begin treatment at the linear accelerator (LINAC), as depicted in Figure S1. This stage involves the precise delivery of radiation therapy according to the plan derived from the initial scan, targeting the tumor while minimizing exposure to surrounding healthy tissue. The use of the LINAC is essential for accurately administering the prescribed radiation doses, which is a critical component of the patient's treatment regimen. However, there is frequently a significant delay between the completion of the RT planning scan and the start of treatment on the LNAC, which can contribute to a misconception in public’s perception. This delay may be interpreted as a lack of urgency or inefficiency in the treatment process, leading to concerns about the quality of care. In reality, this waiting period can be due to various factors, including the high demand for radiotherapy services, the complexity of treatment planning, and the need to ensure that all aspects of the plan are meticulously reviewed and optimized before treatment begins. It is crucial to communicate these factors clearly to patients to manage expectations and provide reassurance about the quality and timing of their care.

Treatment slots on the LINAC are also a secondary rate-limiting step radiotherapy planning process. After the patient undergoes the planning scan, the intricate process of radiotherapy planning is initiated. This process involves multiple interdependent steps and requires the coordinated efforts of a multidisciplinary team, including radiation oncologists, medical physicists, dosimetrists, and radiotherapy technicians. Each professional contributes to developing a precise and individualized treatment plan that maximizes the therapeutic effect while minimizing harm to healthy tissues [4]. Due to the complexity and the high level of coordination required, scheduling treatment slots on the LINAC can be challenging. Limited availability of these slots further exacerbates delays, impacting the timely initiation of treatment. This constraint underscores the importance of efficient workflow management and resource allocation within the radiotherapy department to minimize waiting times and ensure patients receive timely care. This is corroborated by Gensheimer [5], who emphasized that radiotherapy is a complex process with potential for errors. Factors contributing to this complexity include the shortened time between plan approval and the first treatment, the number of prescription items, the involvement of paediatric patients, and the overall complexity of the treatment [6,7].These considerations highlight the critical importance of accounting for both clinical and technological complexities when implementing safety interventions in radiotherapy [5]. Ensuring that these factors are carefully managed is essential to maintaining high standards of patient safety and treatment efficacy.


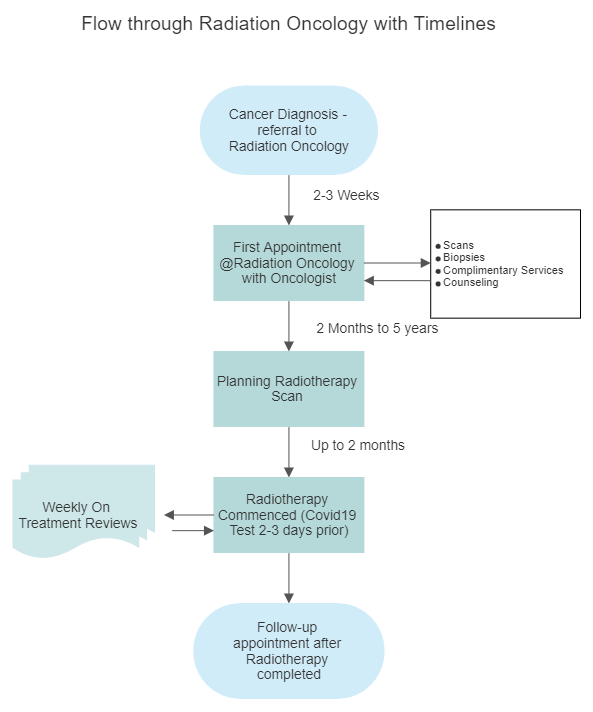


**Figure S1**: **Current workflow through the CMJAH Radiation Oncology Department as illustrated in the patient information pamphlet.**

**Section A2:** **Typical workflow in a radiation oncology department**

The general workflow within radiation oncology is depicted in Figure S2, which begins with an initial consultation, where the radiation oncologist interacts with the patient to discuss the diagnosis and potential treatment modalities. As noted by Korreman et al. [8], this phase involves a thorough review of pertinent medical records and diagnostic exams to assess the patient’s condition accurately. Following the initial consultation, the patient proceeds to a simulation session where advanced imaging technologies, including CT scans, delineate the treatment area. Douglass [9] has indicated that this imaging data is instrumental in creating a detailed 3D representation of the tumour and surrounding tissues. After the simulation, the radiation oncologist collaborates with a planner to contour the tumour and define organs at risk using specialised software tools found in the treatment planning system (TPS). Pasalic et al. [10] have suggested that the objective is to devise an optimised treatment plan that delivers precise radiation doses to the tumour while minimising exposure to healthy tissues.

It is worth mentioning that once the treatment plan is formulated, it is reviewed by the radiation oncologist and medical physicist to ensure its alignment with clinical objectives. Approval of the treatment plan signifies its readiness for implementation. In this case, the approved plan undergoes meticulous quality assurance checks to ascertain its accuracy and safety. As noted by a study conducted by Chera et al. [11], this involves a comprehensive verification process of machine settings, meticulous scrutiny of dose calculations, and tailored patient-specific quality assurance to ensure the plan’s reliability and precision. The patient's position is verified, and then the treatment phase begins, where radiation therapy is administered according to the plan using advanced equipment like LINACs or other specialized systems by radiation therapists [12]. In post-treatment, the patient enters a phase of ongoing care, encompassing regular appointments to monitor treatment response. It has been suggested that continuous surveillance, including follow-up imaging studies and assessments, tracks the tumour’s response to treatment and the patient’s overall health, ensuring meticulous patient care throughout the therapeutic process in radiation oncology [13] [14].

Planning CT Scan

Contouring

Planning

Assessment of plan and approval

Quality assurance

**Figure S2** **Typical workflow implemented in a radiation oncology department**

**Section A3: Radiotherapy treatment process**

The radiotherapy treatment planning process is meticulously sequenced workflow where each step is contingent on the successful completion of the prior step. One critical step of this process is the accurate and timely contouring of target contouring volumes, which serves as a foundation for subsequent planning stages. If the initial phases, particularly target volume contouring are delayed, or if overly optimistic treatment start are set, it can lead to downstream effects and rushed processes. These delays may lead to compromised execution of subsequent steps, such as dose planning and quality assurance. Consequently, this can adversely affect the overall quality of the treatment plan, potentially impact patient outcomes. Ensuring realistic timelines and careful management of each phase is therefore essential to maintain the integrity of the entire radiotherapy process [5]. The first step involves contouring targets like gross tumour volume (GTV) [15] and the OARs [16]. The radiation oncologist prescribes the necessary radiation doses for the targets and sets the maximum doses that organs at risk can receive [16]. Following that, the bottleneck in linear accelerator dosage delivery is often due to the need for more planning radiotherapists/dosimetrists. The planning process is completed by radiotherapists, who can only approve the plan after it is complete. The time between contouring structures and final approval by the radiation oncologist can range from weeks to months, negatively impacting a cancer patient's prognosis. This delay can be addressed by addressing the planning needs of radiotherapists and dosimetrists [17].

**Section A4: Remote therapy planning**

Remote radiotherapy planning represents a transformative approach to modern oncology care, with the potential to significantly reduce treatment backlogs and enhance care delivery. By allowing planning to be conducted at remote sites, this approach not only alleviates the pressure on overburdened facilities but also enhances access to specialized care. With careful consideration of the associated challenges, remote planning can be integrated into existing workflows to create a more efficient, scalable, and patient-centered radiotherapy service. [18]. The Radiation Planning Assistant (RPA) represents a significant advancement in the field of radiotherapy, offering a solution to the challenges of limited access to specialized radiotherapy planning expertise. By automating key components of the planning process, RPA not only enhances the accessibility and efficiency of radiotherapy planning but also ensures consistency and high quality in treatment plans. As a result, patients in remote or resource-limited settings can receive the same level of care traditionally available only in major cancer centers, leading to better outcomes and more equitable cancer care [19]. The RPA has proven its technical effectiveness through robust optimization and feasibility in generating RT plans for cervical, head and neck, and breast cancer. The software’s ability to consistently produce high-quality, clinically viable plans across these diverse and challenging cancer sites underscores its potential to revolutionize radiotherapy planning, making it more accessible and efficient, particularly in remote or resource-constrained environments. These successes affirm RPA's role in advancing the quality of cancer care through automated, yet highly effective, radiotherapy planning solutions [19–21].

Studies comparing RPA-generated plans to those created manually by experienced clinicians have generally shown that RPA can produce plans that are comparable in quality and accuracy. However, there are nuances depending on the specific aspect of the planning process being evaluated. A study has demonstrated that the use of a fully automated RPA for head and neck cancer patients can significantly reduce waiting times, addressing a critical challenge posed by staff shortages. By reducing waiting times and enabling the provision of advanced radiotherapy techniques, RPA technology not only enhances operational efficiency but also broadens access to high-quality cancer care. This development is particularly valuable for clinics with limited resources, as it allows them to offer treatments that were previously beyond their reach, thereby improving patient outcomes and equity in healthcare access [22].

In the case of head and neck cancers, the radiotherapy planning workflow is particularly complex due to the need for precise targeting of the tumor while sparring surrounding critical structures. The use of an automated system like RPA involves several key steps, with a focus on contouring the gross tumor volume and revising auto-generated organs at risks (OARs) and clinical target volumes (CTVs). However, the final refinement and approval by a clinician ensure that the plan meets the high standards required for effective and safe radiotherapy in this challenging anatomical region. This integrated approach leverages the strengths of both automation and human expertise, resulting in optimized treatment plans that can improve patient outcomes. [18]. A study investigating the use of a fully automatic treatment planning system of cervical cancer found that this approach is both effective and potentially a reliable option for low-resource constrained clinics [21]. This finding is particularly significant as it highlights the potential for automated systems to enhance the accessibility and quality of cancer care in settings where resources and specialized expertise are limited. The adoption of such systems could lead to significant improvements in the equity and efficiency of cancer care, ultimately enhancing patient outcomes in low-resource settings.

**References**

1. Mesko S, Weng J, Das P, Koong AC, Herman JM, Elrod-Joplin D, Kerr A, Aloia T, Frenzel J, French KE, Martinez W, Recinos I, Alshaikh A, Daftary U, Moreno AC, Nguyen QN. Using patient flow analysis with real-time patient tracking to optimize radiation oncology consultation visits. BMC Health Services Research BioMed Central; 2022;22(1):1–7. PMID:36514109

2. Akuamoa-Boateng D, Wegen S, Ferdinandus J, Marksteder R, Baues C, Marnitz S. Managing patient flows in radiation oncology during the COVID-19 pandemic: Reworking existing treatment designs to prevent infections at a German hot spot area University Hospital. Strahlentherapie und Onkologie 2020;196(12):1080–1085. PMID:33123776

3. Khan FM, Gibbons JP. Quality of X-ray Beams. Khan’s The Physics of Radiation Therapy. 2014. ISBN:1469881268

4. Slotman BJ, Vos PH. Planning of radiotherapy capacity and productivity. Radiotherapy and Oncology Elsevier Ireland Ltd; 2013;106(2):266–270. PMID:23474286

5. Gensheimer MF, Zeng J, Carlson J, Spady P, Jordan L, Kane G, Ford EC. Influence of planning time and treatment complexity on radiation therapy errors. Practical Radiation Oncology American Society for Radiation Oncology; 2016;6(3):187–193. PMID:26725961

6. Potters L, Kapur A. Implementation of a No Fly safety culture in a multicenter radiation medicine department. Practical Radiation Oncology American Society for Radiation Oncology; 2012;2(1):18–26. doi: 10.1016/j.prro.2011.04.010

7. Walker G V., Johnson J, Edwards T, Gatilao RA, Hayden SE, Riley BA, Sittig DF, Gillin M, Ibbott G, Buchholz TA, Das P. Factors associated with radiation therapy incidents in a large academic institution. Practical Radiation Oncology American Society for Radiation Oncology; 2015;5(1):21–27. PMID:25413430

8. Korreman S, Eriksen JG, Grau C. The changing role of radiation oncology professionals in a world of AI – Just jobs lost – Or a solution to the under-provision of radiotherapy? Clinical and Translational Radiation Oncology Clinical and Translational Radiation Oncology; 2021;26:104–107. doi: 10.1016/j.ctro.2020.04.012

9. Douglass MJJ. Can optical scanning technologies replace CT for 3D printed medical devices in radiation oncology? Journal of Medical Radiation Sciences 2022;69(2):139–142. PMID:35366049

10. Pasalic D, Reddy JP, Edwards T, Pan HY, Smith BD. Implementing an Electronic Data Capture System to Improve Clinical Workflow in a Large Academic Radiation Oncology Practice. JCO Clinical Cancer Informatics 2018;(2):1–12. PMID:30652599

11. Chera BS, Potters L, Marks LB. Restructuring Our Approach to Peer Review: A Critical Need to Improve the Quality and Safety of Radiation Therapy. Practical Radiation Oncology 2020;10(5):321–323. PMID:32888525

12. Marks LB, Adams RD, Pawlicki T, Blumberg AL, Hoopes D, Brundage MD, Fraass BA. Enhancing the role of case-oriented peer review to improve quality and safety in radiation oncology: Executive summary. Practical Radiation Oncology American Society for Radiation Oncology; 2013;3(3):149–156. doi: 10.1016/j.prro.2012.11.010

13. Field M, Hardcastle N, Jameson M, Aherne N, Holloway L. Machine learning applications in radiation oncology. Physics and Imaging in Radiation Oncology Elsevier B.V.; 2021;19(May):13–24. doi: 10.1016/j.phro.2021.05.007

14. Brouwer CL, Dinkla AM, Vandewinckele L, Crijns W, Claessens M, Verellen D, van Elmpt W. Machine learning applications in radiation oncology: Current use and needs to support clinical implementation. Physics and Imaging in Radiation Oncology 2020;16(July):144–148. doi: 10.1016/j.phro.2020.11.002

15. Burnet NG, Thomas SJ, Burton KE, Jefferies SJ. Defining the tumour and target volumes for radiotherapy. Cancer Imaging 2004;4(2):153–161. PMID:18250025

16. Noël G, Antoni D. Organs at risk radiation dose constraints. Cancer/Radiotherapie 2022;26(1–2):59–75. PMID:34953713

17. Physics M. Setting Up a Radiotherapy Programme: Clinical, Medical Physics, Radiation Protection and Safety Aspects. International Atomic Energy Agency 2008;

18. Olanrewaju A, Court LE, Zhang L, Naidoo K, Burger H, Dalvie S, Wetter J, Parkes J, Trauernicht CJ, McCarroll RE, Cardenas C, Peterson CB, Benson KRK, du Toit M, van Reenen R, Beadle BM. Clinical Acceptability of Automated Radiation Treatment Planning for Head and Neck Cancer Using the Radiation Planning Assistant. Practical Radiation Oncology 2021;11(3):177–184. PMID:33640315

19. McGinnis GJ, Ning MS, Beadle BM, Joubert N, Shaw W, Trauernich C, Simonds H, Grover S, Cardenas CE, Court LE, Smith GL. Barriers and Facilitators of Implementing Automated Radiotherapy Planning: A Multisite Survey of Low- and Middle-Income Country Radiation Oncology Providers. JCO Global Oncology 2022;(8):1–10. PMID:35537104

20. Kisling K, Zhang L, Simonds H, Fakie N, Yang J, McCarroll R, Balter P, Burger H, Bogler O, Howell R, Schmeler K, Mejia M, Jhingran A, Court L, Beadle BM. Fully automatic treatment planning for external-beam radiation therapy of locally advanced cervical cancer: A tool for low-resource clinics. Journal of Global Oncology 2019;2019(5):1–8. PMID:30629457

21. Court LE, Kisling K, McCarroll R, Zhang L, Yang J, Simonds H, du Toit M, Trauernicht C, Burger H, Parkes J, Mejia M, Bojador M, Balter P, Branco D, Steinmann A, Baltz G, Gay S, Anderson B, Cardenas C, Jhingran A, Shaitelman S, Bogler O, Schmeller K, Followill D, Howell R, Nelson C, Peterson C, Beadle B. Radiation planning assistant - A streamlined, fully automated radiotherapy treatment planning system. Journal of Visualized Experiments 2018;2018(134):1–9. PMID:29708544

22. Datta NR, Samiei M, Bodis S. Radiation therapy infrastructure and human resources in low- and middle-income countries: Present status and projections for 2020. International Journal of Radiation Oncology Biology Physics Elsevier Inc.; 2014;89(3):448–457. PMID:24751411
